# Digital twin manifesto for the pathology laboratory

**DOI:** 10.1186/s13000-025-01679-2

**Published:** 2025-07-17

**Authors:** Albino Eccher, Fabio Pagni, Massimo Dominici, Luca Reggiani Bonetti, Stefano Marletta, Enrico Munari, Giorgio Cazzaniga, Anil V Parwani, Vincenzo L’Imperio, Angelo Paolo Dei Tos

**Affiliations:** 1 Department of Medical and Surgical Sciences for Children and Adults, University of Modena and Reggio Emilia, University Hospital of Modena, Modena, Italy; 2 Pathology Unit, University Hospital of Modena, Modena, Italy; 3 Department of Medicine and Surgery, Pathology, IRCCS Fondazione San Gerardo dei Tintori, University of Milano-Bicocca, Monza, Italy; 4 Department of Oncology and Hematology, University Hospital of Modena, Modena, Italy; 5 Division of Pathology, Humanitas Istituto Clinico Catanese, Catania, Italy; 6 Department of Diagnostics and Public Health, Section of Pathology, University of Verona, Verona, Italy; 7 Department of Pathology, The Ohio State University Wexner Medical Center, Columbus, OH USA; 8 Surgical Pathology and Cytopathology Unit, Department of Medicine- DIMED, University of Padua School of Medicine, Padua, Italy

**Keywords:** Digital twins, Pathology, Automation

## Abstract

This manuscript presents a manifesto developed by a multifaceted board of stakeholders aimed at guiding the implementation of Digital Twin (DT) technology in pathology laboratories. DTs, already transformative in other sectors, hold substantial promise for enhancing operational efficiency, diagnostic accuracy, and quality of care in pathology. We provide a comparative analysis of traditional versus DT-enhanced workflows across critical steps including accessioning, grossing, processing, embedding, cutting, staining, scanning, diagnosis, and archiving. The framework highlights measurable gains such as up to 90% reduction in labeling errors, 20–30% improvements in slide quality, and 30–50% reductions in diagnostic turnaround time. Alongside these benefits, we address key implementation challenges including upfront infrastructure costs, workforce adaptation, and data security concerns. A practical, phased deployment strategy is proposed—centered on LIS integration, IoT sensors, AI modules, and robust data governance. Estimated setup costs for a medium-sized laboratory range between USD 100,000 and USD 200,000, with a phased rollout timeline of 12–24 months. Supporting technologies like robotic process automation (RPA), collaborative robotics, and edge computing are also discussed as enablers of successful DT adoption. The manifesto closes by identifying critical research gaps, including the need for longitudinal studies evaluating DTs’ clinical and economic impacts, integration within existing hospital IT systems, and ethical implications of AI-assisted diagnostics. Through this collective vision, we provide a realistic and actionable roadmap to drive the transition toward predictive, efficient, and digitally optimized pathology laboratories.

## Introduction

Digital twin technology has emerged as a transformative force across various sectors, facilitating the creation of virtual replicas of physical systems that mimic their real-time behaviors and performances [1–3]. This innovative approach enables organizations to optimize operations, predict failures, and improve decision-making processes through comprehensive data analytics and simulation [4, 5]. This is becoming increasingly valuable in the healthcare setting, where reducing diagnostic errors is crucial to ensuring the most appropriate and timely management of patients, in alignment with the principles of precision medicine [6, 7]. Pathology laboratories are no exception, as they are required to maintain the highest standards of diagnostic accuracy while ensuring rapid turnaround times. However, they continue to face challenges stemming from inefficient procedures and redundant workflow phases, highlighting the urgent need for innovative solutions [8]. While preliminary experiments partly investigated the potentialities of Digital Twins (DTs) in pathology [9], in this manifesto, a multifaceted board of stakeholders envisions how DTs can further extend their benefits within pathology laboratories by introducing alternative operational frameworks, offering virtual tools to enhance laboratory management, and ultimately ensuring the highest levels of diagnostic accuracy for patients.

### Digital twins: from industries to healthcare applications

DTs are virtual representations of physical entities or processes that can be used to simulate, analyze, and optimize operations in real time [10] and are commonly divided into three subtypes: digital twin prototype (DTP), digital twin instance (DTI), and digital twin aggregate (DTA) [11]. The former represents a digital replica of a product before it is actually manufactured (DTP), the second is a digital representation of the single instances of a final product (DTI, e.g. a sensor that collects information from a more complicated machine) and the last is an aggregation of DTIs (DTA). DTs can be conceptualized as a dynamic software model that mirrors the physical counterpart throughout its lifecycle, enabling monitoring, simulation, analysis, and optimization [12]. These DT models found their application in different contexts disrupting the entire product lifecycle management (PLM), from design, to manufacturing, to service and operations. To give an idea of the current and prospective impact of DTs, their global market size has been estimated at USD 16.75 billion in 2023 and is projected to grow at a compound annual growth rate (CAGR) of 35.7% worldwide and of 38.1% in Europe from 2024 to 2030. The healthcare field exemplifies this dynamic, where advanced technologies enable the creation of patient-specific models, continuously adjustable based on tracked health and lifestyle parameters, by translating lean, efficient, and precision-driven methodologies into the realm of precision medicine. Insights from 399 healthcare executives across six countries revealed that the pace of digital transformation within their organization is accelerating (81% of the surveyed executives), mostly pushed with an urgency and call to action from the governance (93%) [13]. This is justified by the rising need of the healthcare executives to adopt a digital-first, people-centric approach across all areas of the organization. Different practical examples already demonstrated the potential impacts of DT implementation in the healthcare setting, ranging from the reduction of up to 10% in sample size for clinical trials to up to 45% improvement in treatment outcomes for radiotherapy planning (Table 1) [14–17]. Early applications of digital technologies in histopathology have shown promising results, particularly through AI models designed to simulate the learning processes of pathologists. Trained on vast repositories of annotated cases, these systems support the interpretation of glass slides by replicating diagnostic patterns and enhancing accuracy across a wide range of conditions [9]. However, DTs can do more for the pathology labs, starting from the critical analysis of workflows from accessioning to final archiving of samples.

**Table 1:** Current examples of the impact of DT implementation within healthcare settings

Field of impact	Setting	DT implementation	Measured outcomes	Reference
Real-time monitoring	Primary Healthcare	Algorithm: MXBoost Sensors: NodeMCU ESP8266 microcontroller Max30102 heart rate and blood oxygen sensor MLX90614 infrared thermometer	Training and testing of vital parameter monitoring reduced of 25% and 33% respectively	[13]
Workflow efficiency	Radiotherapy	Model: DeepProfiler	Up to 45% improvement in treatment outcomes for radiotherapy planning	[14]
Diagnosis	Pathology	Algorithm: vPatho	Diagnostic accuracy in prostate cancer grading in biopsies (k = 0,7) and radical prostatectomies (k = 0,68 )	[9]
Clinical trial (sample size)	Pharmacology	PROCOVA™ (statistical methodology)	Reduction of up to 10% in sample size for future trials (Alzheimer Disease)	[15]
Interventional healthcare	Transversal (oncology, internal medicine, endocrinology, cardiology)	Systematic review (12 studies included)	Increase in 80% of effectiveness across various conditions	[16]

### The pathology lab use case

**Fig. 1:**
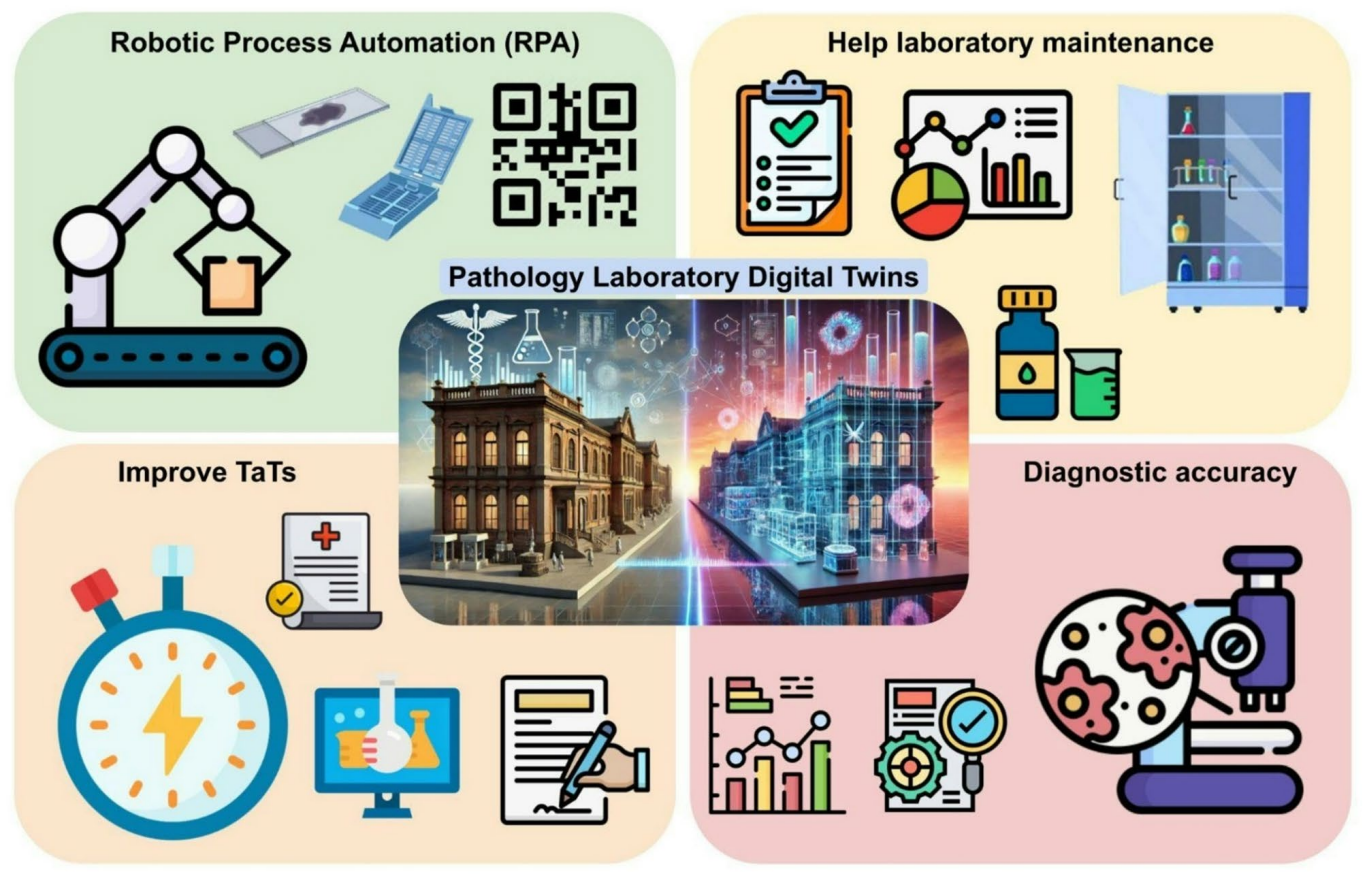
Schematic representation of the potential impact of Digital Twins in the setting of pathology laboratories. Robotic Process Automation (RPA) can ensure high-value creation, fast time-to-value, and notable cost savings, streamlining the pathology department workflow. Digital Twins can help in inventory management and reagent use, helping to minimize waste from expired materials. It can directly influence turnaround times and diagnostic accuracy, as errors can lead to misdiagnosis and inappropriate therapy, which can have detrimental consequences for patients

**Fig. 2:**
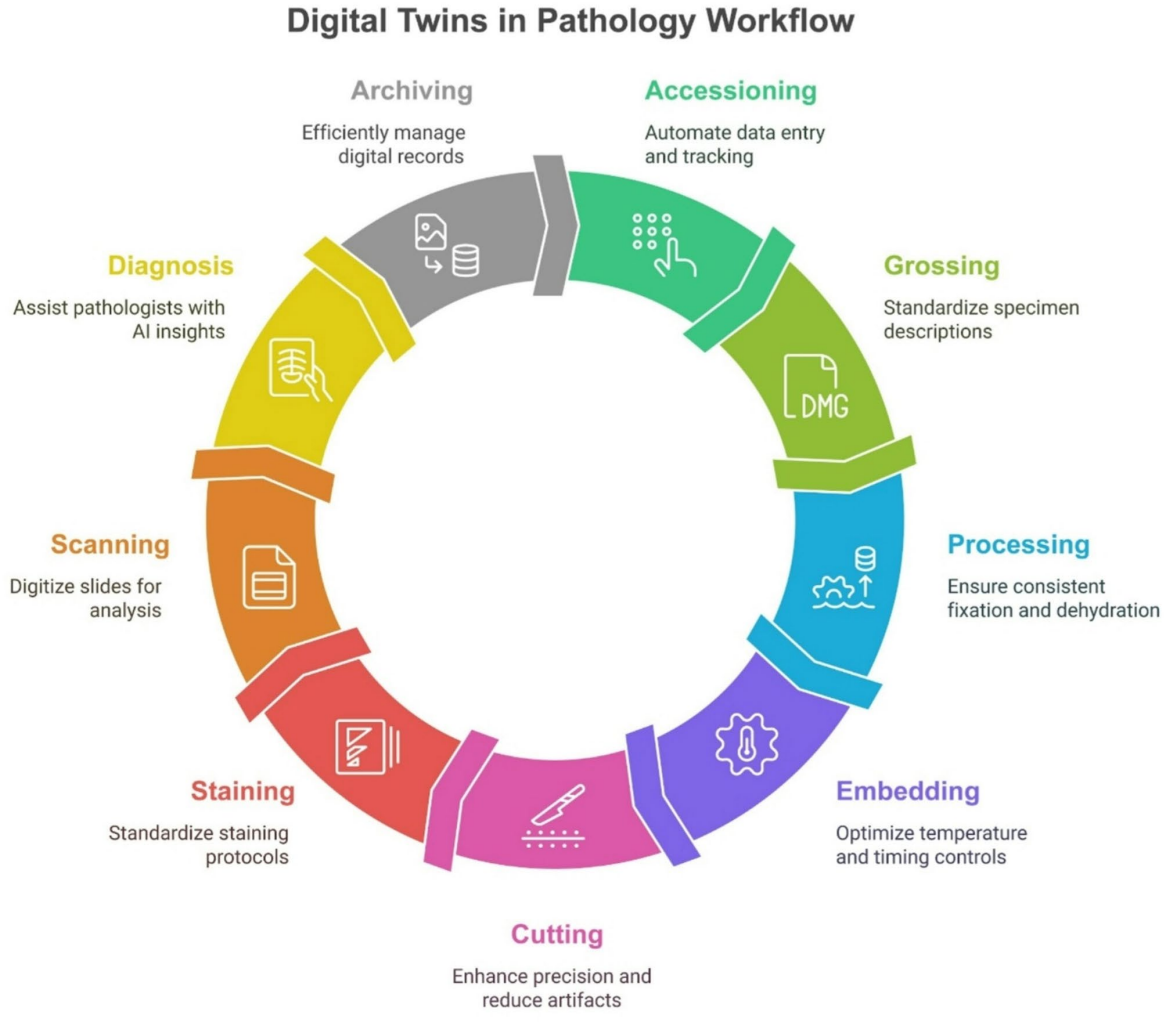
Impact of DTs at every step of the Pathology Laboratory workflow

Pathology laboratories play a critical role in modern healthcare, providing essential diagnostic services. However, these laboratories face numerous challenges, including increasing demand, the need for enhanced accuracy and speed in testing, and the pressures of cost containment [18]. As technological advancements evolve, the adoption of innovative solutions becomes imperative to address these obstacles effectively [19, 20]. The introduction of DTs in pathology laboratories may help stakeholders gain insights into operational efficiencies, identify bottlenecks, and implement solutions to enhance productivity and accuracy (Fig. 1) [21]. However, despite the burgeoning interest in DTs applications in healthcare, several gaps exist in the current literature when it comes to analyse their impact within pathology laboratories. While there are numerous theoretical models for process optimization [22], few empirical investigations explore the practical implementation of these frameworks alongside digital twins in this setting [9]. A comprehensive analysis of laboratory operations both before and after the deployment of this technology is mandatory to highlight potential advancements across several key performance indicators in the pathology workflow, which can be resumed with adequate metrics borrowing from the manufacturing and industry world (Table 2) [7]. The integration of Digital Twin technology into pathology laboratory workflows offers transformative enhancements across all key operational stages—from sample reception to reporting and quality assurance. Several of the performance indicators and optimization approaches employed are derived from established Digital Twin applications in the industrial and logistics sectors, adapted to align with the specific requirements of each phase within the pathology workflow (Fig. 2). In accessioning (sample reception), DTs can provide real-time tracking and routing simulation, reducing labeling errors by up to 90% and enhancing throughput by 15–20%, thereby lowering manual workload and reception bottlenecks. During grossing and processing, DTs may optimize workspace ergonomics, simulate instrument usage, and model reagent conditions, leading to improved tissue sampling accuracy, fewer documentation errors, and a 10–25% reduction in quality issues such as over-/under-processing. Embedding and cutting stages might benefit from predictive simulation and sensor feedback, reducing rework by up to 30% and improving sectioning consistency—translating to better slide quality and up to 20–30% fewer technical errors. In staining, predictive maintenance and digital modeling of reagent dynamics can result in up to a 40% reduction in staining inconsistencies. Scanning workflows are improved through DT-driven workload balancing and image quality validation, increasing scanner uptime and reducing rescans by 25%. As already partly demonstrated [9], diagnostic analysis is notably enhanced through AI-integrated tissue and patient DTs that support lesion progression prediction, pre-screening, and diagnosis validation. This not only augments pathologist accuracy but also reduces diagnostic time by 30–50%, decreasing turnaround time and operational cost. In reporting and archiving, DTs streamline retrieval and storage logistics, forecast usage trends, and ensure regulatory compliance, reducing human error and optimizing space and retrieval time. The digital twin offers a fantastic way to visualize data in real-time and run predictive analytics, helping lab staff spot potential problems before they escalate and cutting down on operational risks. Furthermore, it’s a game changer for inventory management and reagent use, helping to minimize waste from expired materials. With this technology, labs can forecast and predict maintenance, allowing lab managers to anticipate equipment servicing needs based on real-time usage rather than fixed schedules [21, 23, 24]. This reduces unexpected downtime and prevents over- or under-maintenance of critical assets. This not only helps save resources but also trims down costs, boosting the overall economic efficiency of lab operations. From a decision-making perspective, DTs shift control from reactive, human-only judgment to a collaborative model where real-time data and simulations support or automate decisions at critical workflow points (e.g., processing adjustments, re-scanning needs, diagnostic flags). Dynamic feedback loops are a core strength of DTs: real-time performance metrics are continuously fed into the system to adapt parameters, flag anomalies, and predict failures before they occur—enhancing both speed and reliability. This evolution also transforms staff roles, introducing new responsibilities in DT system oversight, data interpretation, and AI collaboration, while reducing repetitive manual tasks. Pathology technologists and lab managers may take on hybrid roles involving both technical execution and digital system calibration.

**Table 2:** Actionable points within the pathology laboratory workflow that can benefit from the introduction of digital twins. *some of these impact measures are borrowed from industry and logistics DT applications close to the corresponding workflow phase

Path Lab workflow step	Current workflow	DT implementation benefits	Potential DT impact*
Accessioning	- Order entry generated at the creation of the case (sampling room) - Case accessed within Path Lab with LIS using order entry - Internal progressive case ID attributed to accessioned case	- Real-time monitoring of specimen entry and traceability - Simulation of specimen routing based on priority, volume, and lab load - Detection of bottlenecks and labeling mismatches before they propagate-	- Reduction in mislabeling errors by up to 90% (as observed in logistics DT applications). - Faster specimen intake workflow (~ 15–20% gain in throughput).
Grossing	- Identification of the case through ID (barcode reader) - Macroscopic description and sampling of the specimen - Cassette creation and eventual photographic documentation	- Virtual simulation of gross room layouts to optimize workspace and ergonomics - Feedback on instrument usage, specimen tracking, and cutting guidance via AI-enhanced DTs - Integration with voice-assisted systems for accurate, real-time documentation	- Improved accuracy in tissue sampling - Reduction in dictation/documentation errors
Processing	- Automatic processing of the cassettes to be embedded in the following step - Possible checkpoint for identity of in and out cassettes from grossing to processing - Possible diversification of processing protocols (e.g. small vs. larger samples)	- Modeling of processing parameters (e.g., reagent conditions, timing) to predict outcomes - Preemptive alerts if protocols deviate from expected digital twin profile	- Reduction in under/over-processing artifacts - Standardization of tissue quality across batches (~ 10–25% fewer repeat blocks)
Embedding	- Manual, automated or semi-automated embedding of the cassette content with paraffin - Possible checkpoint for the assessment of cassette batch completeness	- Digital twin tracking of cassettes to verify orientation and completeness - Simulation of tissue placement to prevent sample loss or orientation errors	- Reduced embedding errors and need for re-embedding (~ 30% fewer remakes)
Cutting	- Progressive sectioning of the block to obtain adequate and representative sections to be placed on a glass slide for staining - Barcode-mediated identification of the FFPE block and consequent glass slide creation - Delicate and time-consuming trimming step	- Monitoring blade wear and section thickness in real-time - Guidance for optimal sectioning parameters based on tissue type	− 20–30% improvement in slide quality - Reduction in technician error due to fatigue or technique inconsistency
Staining	- Automatic staining of batches with histo or immunohistochemistry stainers from different vendors - Manual reagent supply	- Modeling stain quality based on reagent concentration, temperature, and time - Predictive maintenance for staining instruments	- Improved staining consistency and color fidelity - Up to 40% reduction in staining errors
Scanning	- Manual or facilitated scanner loading - Optional continuous loading depending on the scanner model	- Optimization of scanner workload, prediction of scan time, and preemptive detection of scan failures - Image quality validation using trained digital twin models	- Improved scanner uptime and reduced rescan rates (~ 25% gain) - Accelerated turnaround time for digital workflows
Diagnosis	− 100% pathologist based, under the microscopy or with digital slides	- Creation of digital patient and tissue twins to assist in diagnostic simulation - Predictive models for lesion progression, diagnosis validation, or treatment selection	- Augmented diagnostic accuracy. - Enhanced pathologist efficiency via AI-supported pre-screening (e.g., 30–50% time saved in some trials)
Archiving	- Manual archiving of residual material from grossed/sampled specimens, tissue blocks and glass slides, in ordered way - Time consuming and tedious task	- Tracking specimen life cycle and optimizing storage logistics using virtual replicas - Forecasting usage patterns for efficient retrieval and regulatory compliance	- Reduced retrieval times - Lower archival error rate and space optimization

### Facilitating technology: from robotics to AI

The projected growth of digital twin (DT) technologies is largely driven by the increasing adoption of Industry 4.0 frameworks and advancements in enabling technologies such as big data analytics, the Internet of Things (IoT), artificial intelligence (AI), and machine learning (ML). The expanding integration of IoT and big data is particularly influential, as businesses seek to operate more efficiently, streamline workflows, and reduce time to market. Concurrent developments in virtual reality (VR) and augmented reality (AR) are also accelerating DT evolution. Organizations are increasingly using IoT and AI to collect and analyze data from connected devices, which can be integrated into digital twin models to reflect real-time operations and performance. This supports enhanced monitoring, early detection of performance issues, and predictive maintenance. The growing demand for digital twin solutions is prompting industry players to expand product portfolios and extend their global presence to capture new market opportunities. In the specific context of pathology laboratories, emerging technologies such as Robotic Process Automation (RPA) are beginning to reshape operational frameworks [18]. Known for its speed, efficiency, and cost-saving potential, RPA is one of the fastest-growing components in DT platforms. Pathology labs, which traditionally rely on labor-intensive workflows requiring high flexibility, face increasing pressure to manage large sample volumes effectively. Automation—ranging from lean automation to hybrid human-machine systems—can bridge this gap, enhancing productivity while maintaining flexibility [25]. The advent of Robotics 2.0 and collaborative robots (cobots) is also transforming laboratory environments. Cobots, equipped with advanced safety and motion capabilities, offer adaptable and efficient solutions for manual tasks. Their integration into laboratory settings fosters human-robot collaboration and is driving investment in R&D and advanced manufacturing, further stimulating demand for DT platforms. In addition, DTs enable distributed remote control of laboratory assets, which introduces increased demands on secure IoT identity management, authentication, and authorization systems. As DTs become more embedded in healthcare infrastructure, their role in simulating and optimizing medical device performance will become increasingly critical [26].

### Challenges and potential solutions

The implementation of DTs in pathology laboratories presents several domain-specific challenges that must be carefully addressed to enable successful integration and long-term sustainability. Among the most significant barriers are upfront costs, staff resistance to technological change, and concerns around data privacy and interoperability. Initial investment in DT infrastructure—including data acquisition systems, real-time integration platforms, and computational resources—can be limiting, especially for smaller laboratories or institutions with budget constraints. Phased deployment models may be a solution with gradual integration of DT components starting from high-impact workflow segments (e.g., specimen tracking or quality control monitoring), with scalability toward full-system modeling over time [27]. Staff resistance is another common hurdle, often driven by unfamiliarity with DT technologies and perceived disruption to established workflows. To address this, targeted staff training modules and continuous professional development programs can improve user acceptance and ensure that technical personnel are equipped with the necessary skills to engage with DT systems effectively [28]. Early involvement of key stakeholders in the design and testing phases can also enhance buy-in and reduce resistance [29]. Data privacy and security remain central concerns, particularly given the sensitive nature of patient data managed in pathology settings. Solutions should include robust data governance frameworks, with clear protocols for anonymization, access control, and compliance with standards such as GDPR or HIPAA [30]. Employing edge computing or hybrid cloud architectures may also reduce data exposure by keeping critical processing on-premises while leveraging external computation when appropriate [31]. From a practical integration standpoint, a hybrid strategy appears to be the most feasible and sustainable route for deploying DTs in pathology laboratories. This approach supports the adaptation of DT functionalities within existing Laboratory Information Systems (LIS), rather than requiring complete infrastructure overhaul [32].

### Future perspectives and research gaps

Transitioning to Digital Twin (DT) technology in pathology laboratories requires a phased, practical roadmap involving infrastructure setup, AI integration, staff training, and data governance. Initial investments include IoT sensors, computing systems, and software platforms, with estimated costs ranging from USD 100,000 to USD 200,000 depending on lab size and scope [33]. Staff training and change management are essential to ensure adoption, alongside robust data privacy protocols compliant with GDPR or HIPAA. A 12–24 month phased implementation is recommended, beginning with high-impact areas [34]. Future research should focus on long-term clinical and economic outcomes, integration with existing LIS systems, and ethical implications of AI-driven diagnostics. With continued collaboration among labs, tech providers, and academia, DTs have the potential to transform pathology into a predictive, data-driven discipline.

## Conclusions

This manifesto supports a phased, LIS-centric approach to implementing Digital Twins (DTs) in pathology, combining technical integration, staff training, and data governance. Comparative insights suggest DTs can reduce labeling errors by up to 90%, improve slide quality by 20–30%, and cut diagnostic turnaround times by 30–50%, ultimately enhancing efficiency, accuracy, and patient care.

## Data Availability

No datasets were generated or analysed during the current study.
